# Use of a New Non-Pyrophoric Liquid Aluminum Precursor for Atomic Layer Deposition

**DOI:** 10.3390/ma12091429

**Published:** 2019-05-02

**Authors:** Xueming Xia, Alaric Taylor, Yifan Zhao, Stefan Guldin, Chris Blackman

**Affiliations:** 1 Department of Chemistry, University College London, 20 Gordon Street, London WC1H 0AJ, UK; xueming.xia.16@ucl.ac.uk; 2 Department of Chemical Engineering, University College London, Torrington Place, London WC1E 7JE, UK; alaric.taylor@ucl.ac.uk (A.T.); s.guldin@ucl.ac.uk (S.G.); 3 Department of Life Sciences, Imperial College London, South Kensington Campus, London SW7 2AZ, UK; yifan.zhao15@imperial.ac.uk

**Keywords:** chemical vapor deposition, atomic layer deposition, aluminum oxide, aluminum tri-sec-butoxide, thin film

## Abstract

An Al_2_O_3_ thin film has been grown by vapor deposition using different Al precursors. The most commonly used precursor is trimethylaluminum, which is highly reactive and pyrophoric. In the purpose of searching for a more ideal Al source, the non-pyrophoric aluminum tri-sec-butoxide ([Al(O^s^Bu)_3_], ATSB) was introduced as a novel precursor for atomic layer deposition (ALD). After demonstrating the deposition of Al_2_O_3_ via chemical vapor deposition (CVD) and ‘pulsed CVD’ routes, the use of ATSB in an atomic layer deposition (ALD)-like process was investigated and optimized to achieve self-limiting growth. The films were characterized using spectral reflectance, ellipsometry and UV-Vis before their composition was studied. The growth rate of Al_2_O_3_ via the ALD-like process was consistently 0.12 nm/cycle on glass, silicon and quartz substrates under the optimized conditions. Scanning electron microscopy and transmission electron microscopy images of the ALD-deposited Al_2_O_3_ films deposited on complex nanostructures demonstrated the conformity, uniformity and good thickness control of these films, suggesting a potential of being used as the protection layer in photoelectrochemical water splitting.

## 1. Introduction

As a dielectric metal oxide, Al_2_O_3_ exhibits a high transparency, large bandgap and excellent electrical insulation properties, hence it is widely applied in electronic devices and electrochemistry, e.g., as a protection layer [1,2,3,4,5,6]. Commonly used methods for making Al_2_O_3_ films include sol-gel, sputtering, evaporation, physical vapor deposition (PVD), chemical vapor deposition (CVD) and atomic layer deposition (ALD) [7,8,9,10,11]. CVD is a widely used thin film deposition technique to provide good quality thin films with control over the chemical composition. ALD uses sequential pulses of precursors which react with the surface in a self-limiting way [12,13], giving better control of material thickness, stoichiometry and conformality than normally afforded via CVD or PVD [14,15].

**Figure 1:**
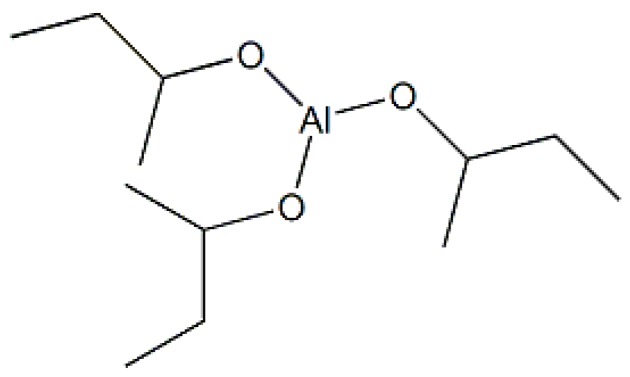
The structural formulas of aluminum tri-sec-butoxide.

Careful consideration must be exercised in precursor selection for all vapor deposition processes. Whilst they may be gas, liquid or solid at room temperature, they must be volatile and thermally stable under zationvaporization conditions. Widely used oxygen-containing precursors are H_2_O, O_2_ and O_3_, whilst precursors for metals include metal halides, metal alkyls, metal alkoxides, etc [16]. Typical precursors for Al include aluminum chloride [AlCl_3_] [17], trimethylaluminum [TMA, Al(CH_3_)_3_] [18], aluminum tri-isopropoxide [AIP, Al(O^i^Pr)_3_] [19] and aluminum acetylacetonate [Al(acac)_3_] [20]. Among the above, TMA is the most commonly used Al precursor, but it is highly reactive, moisture sensitive and pyrophoric, which makes handling inconvenient. The most common non-pyrophoric Al precursor is Al(O^i^Pr)_3_, but as a solid precursor there is significant possibility of condenzation during vapor transport leading to blockages and consequent reactor downtime. Therefore, in this study, the suitability of aluminum tri-sec-butoxide (ATSB, [Al(O^s^Bu)_3_], [Al(OCH(CH_3_)CH_2_CH_3_)_3_] was investigated as an Al precursor. At room temperature, ATSB is a non-pyrophoric liquid which contains no halogens atoms (Figure 1). ATSB has been previously reported as a metal-organic chemical vapor deposition (MOCVD) precursor [21]. However, to the best of our knowledge, it has never been studied for use in ALD before.

In this research, ATSB was used in single-source CVD (also in ‘pulsed CVD’ mode) and ALD to deposit Al_2_O_3_ thin films. The deposition parameters (precursor temperature, deposition temperature, carrier gas flow rate, pulse time and purge time) were optimized on different substrates to demonstrate controllable and uniform film deposition.

## 2. Materials and Methods

### 2.1. Chemicals and Materials

Aluminum tri-sec-butoxide (ATSB, 97%) was purchased from Sigma-Aldrich (St. Louis, MI, USA). Propan-2-ol (laboratory reagent grade), methanol (HPLC grade) and ethanol absolute (HPLC grade) were received from Fisher Scientific (Loughborough, UK). Super premium glass microscope slides were purchased from VWR International, LLC (Radnor, PA, USA). Quartz slides were obtained from Wuxi Crystal and Optical Instrument Co., Ltd (Wuxi, China). P-doped silicon wafers were bought from Suzhou Crystal Silicon Electronics & Technology Co., Ltd (Suzhou, China). Deionized water was obtained from ELGA Purelab Option (ELGA LabWater). Liquid nitrogen and argon gas were provided by the BOC group (Surrey, UK).

### 2.2. Al_2_O_3_ Thin Film Deposition via CVD

Aluminum tri-sec-butoxide (maintained at 120 °C) was used as a single-source CVD precursor. ATSB vapor was carried to the reaction chamber via Ar carrier gas. To operate in CVD mode, the inlet valve and outlet valve of the ATSB bubbler were kept open for the entire deposition and no water co-reactant was used. The ATSB bubbler was heated to 115 °C, 120 °C, 125 °C, respectively. The gas flow rate was set to 150 sccm. The substrate was heated to 300 °C, 350 °C or 400 °C for film deposition. Each deposition took 24 hours. A deposition mode called ‘pulsed CVD’ was used to mimic the metal precursor half cycle in ALD. A cycle in pulsed CVD mode only contained one ATSB pulse (20 s or 1 min) and one argon purge (1 min). For all experiments, the pipework was heated to 170 °C to prevent precursor condenzation.

### 2.3. Al_2_O_3_ Thin Film Deposition via ALD

Al_2_O_3_ films were grown via ALD between 250 and 1000 deposition cycles. ATSB (maintained at 120 °C) and deionized water (maintained at 5 °C) were held in bubblers as precursors and the precursor vapor was carried to the reaction chamber via Ar carrier gas. Each cycle consisted of an ATSB pulse (2.5–20 s), an argon purge (1–3 min), then followed by a 2 s water vapor pulse and an argon purge of at least 3 min. It is worth noting that the minimum dose time in this reactor is 2 s, which inevitably requires longer purge times than normally observed in an ALD reaction. The gas flow rate through the ATSB bubbler and through the purge line was set to 120 sccm. The substrate was heated to 200 °C, 250 °C, 300 °C or 350 °C for film deposition, respectively. Glass, quartz and silicon substrates were used for the comparative study of film growth and quality. Al_2_O_3_ films of different thickness (20–300 cycles) were also deposited on Au NPs/WO_3_ NRs system to demonstrate uniformity.

### 2.4. Characterization

The thickness of the films was measured using a Variable Angle Spectroscopic Ellipsometer (VASE, SEMILAB SE-2000, Semilab, Budapest, Hungary) in the wavelength range of 1.25–5 eV with variable measuring angle (56°, 57°, 58°, 59° and 60°). The optical model used for ellipsometric characterization of the ALD-like thin films was an effective medium approximation (EMA) comprising of air and the aluminum oxide (previously reported refractive index) with variable concentration [22,23]. Glass substrates were modelled using standard parameters for sodlime glass and the silicon substrates were assumed to have a 1 nm native oxide. Fittings of the ellipsometric data converged without the need for substrate-film and film-air diffusion layers. The crystalline structure of the samples was examined by X-ray diffraction (XRD, Bruker, Billerica, MA, USA, D8 Discover LynxEye) with a current of 40 mA and a voltage of 40 kV. The data was collected with a scanning rate of 0.05°/s over a 2θ range from 10° to 66° counted at 2 s per step. X-ray photoelectron spectroscopy (XPS, Thermo Fisher Scientific, Waltham, MA, USA, Neslab ThermoFlex1400) with a monochromatic Al Kα X-ray source was used to measure the elemental composition and electronic states of the elements. Tapping mode atomic force microscopy (AFM, Bruker, Billerica, MA, USA, Dimension FastScan2-SYS) using a NCLR tip was employed to investigate the surface structure of the sample. The morphologies of the samples were observed by field emission scanning electron microscope (FESEM, JEOL, Tokyo, Japan, JSM-7800F) with a current of 10 mA and an acceleration voltage of 5–15 kV after Au coating. Films deposited on Au/WO_3_ films and were examined by transmission electron microscopy (TEM, JEOL, Tokyo, Japan, JEM-2100) after they were sonicated in methanol and dropped on copper grids to show the details. Photoelectrochemical (PEC) measurement was carried out using a homebuilt PEC system with a Xe lamp (75W, USHIO, California, USA) equipped with an AM 1.5 G filter (Newport, California, USA) using a Pt mesh counter electrode, a Ag/AgCl reference electrode and Na_2_SO_4_ electrolyte. All the photoelectrochemical tests were performed using a potentiostat (Interface 1000, Gamry, Pennsylvania, USA).

## 3. Results and Discussion

### 3.1. Deposition Parameter Optimization for CVD

Aluminum sec-butoxide ([Al(O^s^Bu)_3_]) is a high viscosity liquid metal alkoxide, typically preferred for CVD reactions in comparison to solid precursors such as Al(O^i^Pr)_3_. The vapor pressure of ATSB at different temperatures was calculated based on data from literature [24,25] (Figure S1). Note that the function of vapor pressure in the Antoine equation is fitted as lg(PTorr) = 10.16 − 4177.25/T.

According to our previous study [26], a metal precursor vapor pressure higher than 0.13 Torr would generate enough metal precursor vapor in our reactor for one pulse. Therefore, a temperature above 115 °C would provide sufficient vapor pressure.

**Figure 2:**
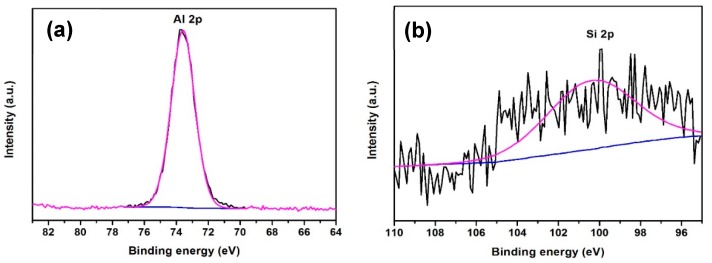
High resolution XPS spectra of (**a**) Al 2p and (**b**) Si 2p of Al_2_O_3_ film deposited on glass via CVD.

Initial experiments focused on optimizing the precursor temperature, gas flow rate and deposition temperature for CVD deposition. After several attempts at 115 °C, 120 °C and 125 °C, the optimum ATSB bubbler temperature was found to be 120 °C. Meanwhile, increasing the precursor temperature further did not produce any significant increase in thickness at a deposition temperature of 350 °C (additional precursor not utilised in deposition). The composition of the films was analyzed by XRD, Raman spectroscopy (inVia Raman microscope, Renishaw, Gloucestershire, UK) and XPS. The crystallization temperature of γ-Al_2_O_3_ is at least 600 °C and 1000 °C is needed for it to transform to α-Al_2_O_3_ [27,28,29]. As the deposition temperature here was much lower than the crystallization temperature of Al_2_O_3_, it is consistent that neither XRD patterns nor Raman spectra showed pronounced peaks (Figures S2 and S3). For a film deposited at 400 °C for 24 h with 120 °C ATSB precursor temperature and 150 sccm gas flow, the Al 2p spectrum (Figure 2a) displayed an intense peak for the Al 2p ionization at 73.6 eV which matches with the binding energy of Al^3+^ in Al_2_O_3_ [30]. The Si 2p peak is of very low intensity (Figure 2b), which indicates that no Si from the glass substrate is observed, thus the Al_2_O_3_ film covers the entire area of the analysis spot. These findings are consistent with the films deposited via CVD using ATSB as a single-source precursor as an amorphous Al_2_O_3_.

**Figure 3:**
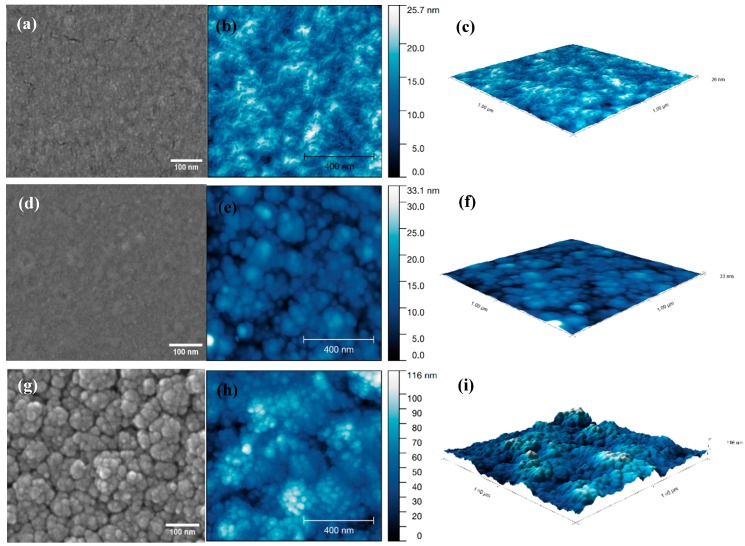
SEM images, AFM scans and 3D images of Al_2_O_3_ films deposited for 24 h with 150 sccm Ar gas flow, precursor temperature at 120 °C and deposition temperature at (**a**–**c**) 300 °C; (**d**–**f**) 350 °C; (**g**–**i**) 400 °C.

To observe the effect of substrate temperature, the CVD deposition temperatures were set to 300 °C, 350 °C and 400 °C (with a bubbler temperature of 125 °C). AFM scans (Figure 3) show an increase in the roughness of film surface as deposition temperature increases; R_q (300 °C)_ = 3.45 nm, R_q (350 °C)_ = 3.76 nm and R_q (400 °C)_ = 18.21 nm, which can also be seen in SEM images (Figure 3). Increasing the deposition temperature increases film roughness, which is typically unfavorable for protective coatings. Consequently, we concluded that 350 °C provided a good balance between growth rate and surface roughness.

### 3.2. Pulsed CVD

**Figure 4:**
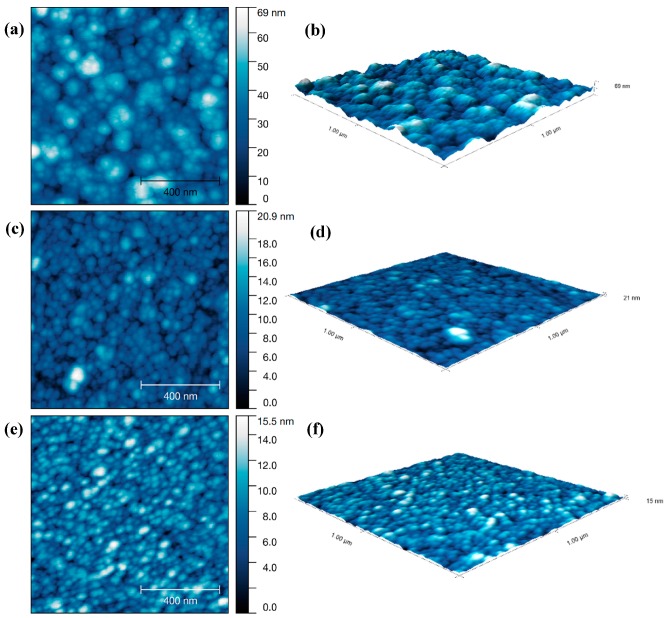
AFM scans and 3D images of Al_2_O_3_ films deposited at 350°C via (**a**,**b**) continuous CVD; (**c**,**d**) pulsed CVD (1 min ATSB pulse) and (**e**,**f**) pulsed CVD (20 s ATSB pulse).

After the optimization of CVD parameters, a pulsed CVD process was introduced to identify the minimum duration required for a metal precursor pulse, thereby preventing over-dosing. In order to obtain vapor transport (film growth), the pulse of ATSB was set to 20 s or 1 min, the deposition temperature was 350 °C and the precursor temperature was fixed at 120 °C with an Ar flow rate of 120 sccm. Comparing the AFM images of Al_2_O_3_ films deposited via continuous CVD (24 h), pulse CVD (1 min ATSB pulse, 1 min Ar purge, 1000 cycles) and pulsed CVD (20 s ATSB pulse, 1 min Ar purge, 1000 cycles). Figure 4 shows the surface roughness decrease from R_q (continuous deposition)_ = 10.19 nm to R_q (1min pulse)_ = 2.51 nm and R_q (20 s pulse)_ = 2.27 nm. We concluded from these data that a 20 s pulse produced complete coverage and was therefore a suitable starting point for ATSB precursor dosing in an ALD process.

### 3.3. Deposition Parameter Optimization for ALD

The reaction mechanism when using ATSB and water as ALD precursors may be expected as [31,32]:

x –OH (s) + Al(O^s^Bu)_3_ (g) → (–O–)*_x_*Al(O^s^Bu)_3−*x*_ (s) + *x*^s^BuOH (g) (1)

(–O–)*_x_*Al(O^s^Bu)_3−*x*_ (s) + (3−*x*) H_2_O (g) → (–O–)*_x_*Al(OH)_3−*x*_ (s) + (3−*x*) ^s^BuOH (g) (2)

with *x* = 1 being the monodentate, *x* = 2, bidentate and *x* = 3, tridentate, respectively, depending on the number of alkoxide ligands.

A full ALD reaction contains two half reactions. In the first half reaction, ATSB reacts with OH groups on the substrate surface to produce (–O–)*_x_*Al(O^s^Bu)_3−*x*_, where x is the number of OH surface sites react with ATSB which also determines the geometrical configuration on the surface. The (–O–)*_x_*Al(O^s^Bu)_3−*x*_ group can react with H_2_O to form (–O–)*_x_*Al(OH)_3−*x*_ in the second half of the reaction, therefore, the surface layer is covered with OH groups again, allowing the first half reaction to take place again [33].

**Figure 5:**
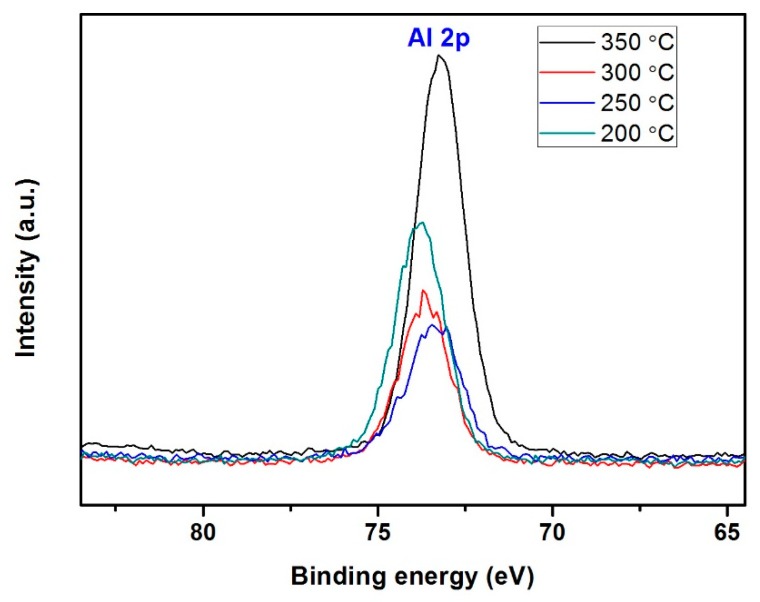
High resolution XPS spectra of Al elements under different deposition temperature, 200 °C, 250 °C, 300 °C and 350 °C.

The deposition temperature used in ALD should be lower than that used in CVD to avoid uncontrolled CVD-like film growth, therefore the ALD deposition temperature was decreased in comparison to CVD in increments from 350 °C to 300 °C, 250 °C and 200 °C whilst the other parameters were kept unchanged (20 s ATSB pulse, 1 min Ar purge, 2 s H_2_O pulse and 3 min Ar purge with 120 sccm Ar gas flow deposited for 500 cycles). XPS spectra of this series of samples indicate that films deposited at 200 °C, 250 °C and 300 °C show similar binding energy intensity at approximately 74 eV (Figure 5). The growth rate of Al_2_O_3_ was between 1.9–2.3 nm/cycle in the deposition temperature range of 200 °C–300 °C with a 20 s pulse time, thus 200 °C was selected in further experiments (Figure S4). The thickness and growth rate were found to be much higher than previously reported values (0.1 nm/cycle) [34,35,36,37,38,39] indicating a combination of ALD and CVD-like growth. One likely explanation is that the dose time of ATSB was too long for the purge time used, therefore the ATSB dose time was reduced from 20 s to 2.5 s with the aim to avoid CVD-like growth. Changing the ATSB purge time from 1 min to 3 min reduced the deposition rate, while more than 3 min for a 2.5 s dose time resulted in no change in the growth rate, therefore 3 min purge was considered optimum for a 2.5 s Similarly, for a 2 s H_2_O dose, changing the purge time from 3 min to 5 min had no effect, indicating that a 3 min purge was sufficient to ensure all water was evacuated between cycles.

With a 2.5 s dose time, the thickness reduced to approx. 50 nm after 500 cycles, which gave growth rates in the range of 0.12 to 0.15 nm/cycle with successful deposition on silicon, quartz and glass substrates (Table S1). The growth rate using ATSB as an ALD precursor is similar to that using TMA or AlCl_3_ (0.08–0.2 nm/cycle) [38,40,41,42]. Thus, the ALD-like parameters were fixed at an ATSB bubbler temperature of 120 °C, deposition temperature of 200 °C and gas flow rate of 120 sccm (a full cycle includes 2.5 s ATSB pulse, 3 min Ar purge, 2 s H_2_O pulse and 3 min Ar purge).

**Figure 6:**
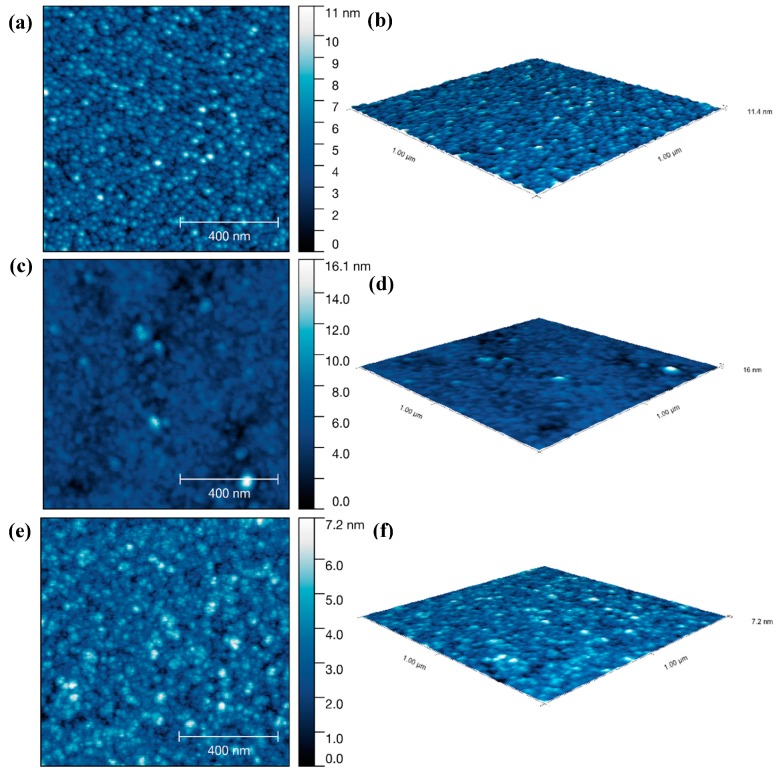
AFM scans and 3D images of Al_2_O_3_ films after 250 ALD cycles, on (**a**,**b**) silicon; (**c**,**d**) quartz; (**e**,**f**) glass. A cycle includes 2.5 s ATSB pulse, 3 min Ar purge, 2 s H_2_O pulse and 3 min Ar purge, deposition temperature 200 °C, gas flow rate 120 sccm.

The density of the Al_2_O_3_ component in the ellipsometric EMA model varied between 0.720 and 0.757 (Table S1 and Figure S5). The films deposited with short 2.5 s ATSB pulse times showed lower growth rates (0.12–0.15 nm/cycle) and higher densities than those grown with longer 20 s pulses. The root mean square roughness of Al_2_O_3_ film on three substrates were R_q(silicon)_ = 1.48 nm, R_q(quartz)_ = 1.16 nm and R_q(glass)_ = 0.96 nm, suggesting an applicability of Al_2_O_3_ on a variety of substrates (Figure 6).

### 3.4. ALD Al_2_O_3_ Films on Complex Structured Au/WO_3_

Our aim is to use this ALD Al_2_O_3_ film as a passivation layer for photoelectrode. In former studies of ALD thin films that include Al_2_O_3_ and other oxides [43,44,45], it has been mentioned that either an ultrathin film of several nanometers or a thin film with a thickness above 20 nm showed different degrees of current density enhancement during a photoelectrochemical test.

**Figure 7:**
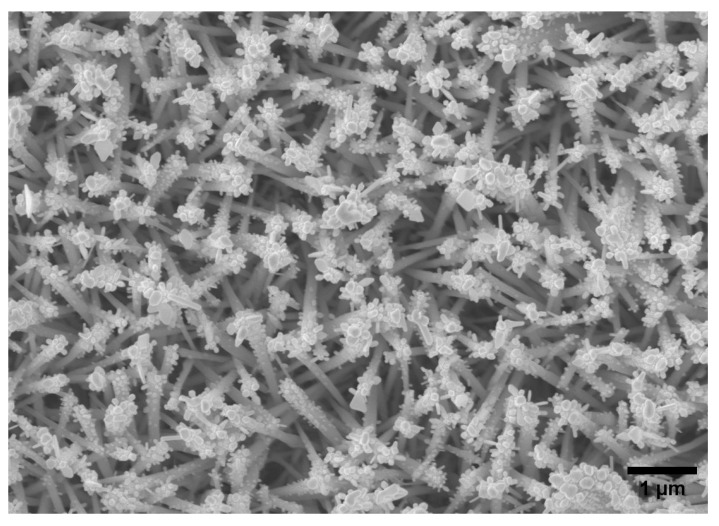
SEM image of Au/WO_3_ film deposited via CVD (top view).

**Figure 8:**
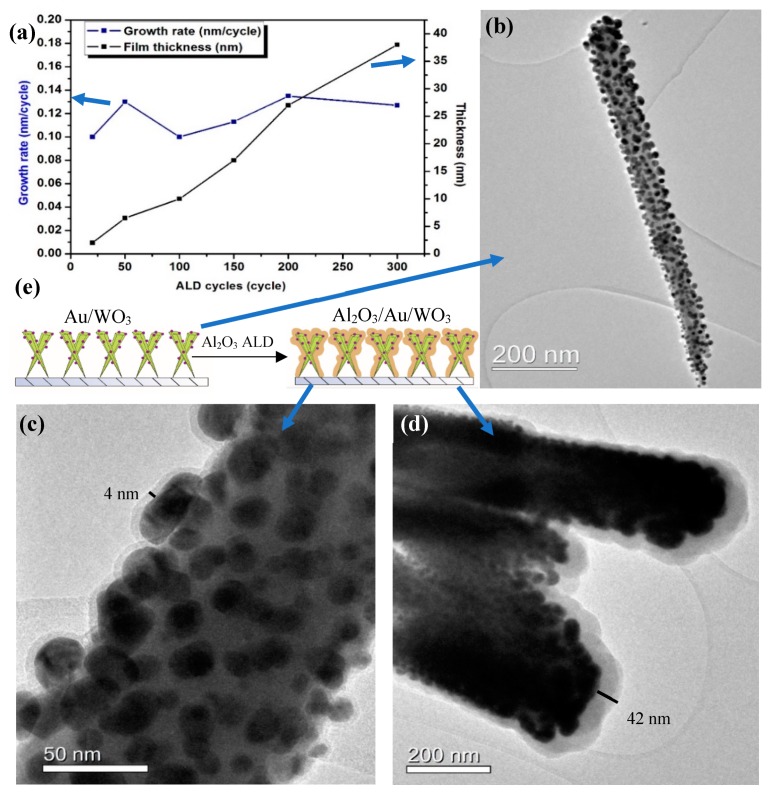
(**a**) Al_2_O_3_ film growth rate (blue) and film thickness (black) as a function of reaction cycles, (**b**) Au/WO_3_ structure, (**c**) Au/WO_3_ with 50 cycles ALD Al_2_O_3_ growth on top, (**d**) Au/WO_3_ with 300 cycles ALD Al_2_O_3_ growth on top, (**e**) Schematic procedure of ALD Al_2_O_3_ growth.

Al_2_O_3_ films were deposited via the ALD-like process on a complex nanostructure consisting of Au nanoparticles on needle-like WO_3_ nanostructures (Au/WO_3_, Figure 7 and Figure 8). Au/WO_3_ is an electrode prepared in house on an FTO glass for water splitting. However, it is not stable in any kind of electrolyte. Therefore, Al_2_O_3_ films were deposited with the aim to protect the electrode without severely affecting the photocurrent required for water splitting. The SEM image of the Au/WO_3_ shows a plane of nanoneedles which has a high surface area and high porosity. Due to the complex structure, the thickness of the ALD layer was difficult to measure using an optical method, therefore film thickness during deposition was recorded using a ‘witness’ planar substrate inserted in the reactor at the same time with the sample. Growth rate as a function of number of cycles was plotted (Figure 8a). The growth rate of Al_2_O_3_ on both the witness plate and the nanostructures was between 0.1 nm/cycle and 0.13 nm/cycle (shown in Figure S6). For a given number of cycles, similar thickness were achieved on both surfaces. There is a strong correlation between the film thickness and the number of ALD cycles (Table 1), e.g., 50 cycles and 300 cycles gave 4 nm and 40 nm respectively on both the planar substrate (measured using ellipsometry) and the complex nanomaterial architecture (measured by TEM, Figure 8c,d).

**Figure 9:**
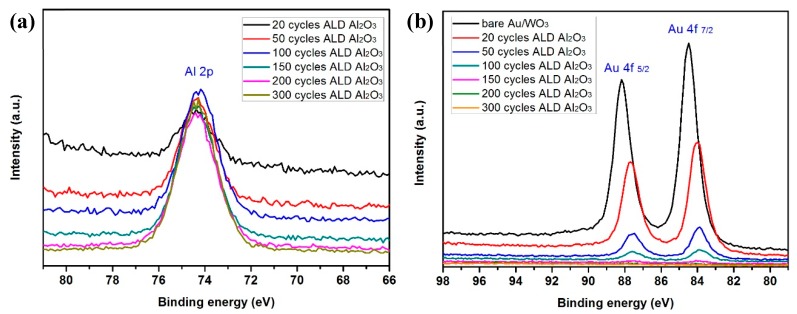
XPS spectra of (**a**) Al 2p and (**b**) Au 4f in Al_2_O_3_/Au/WO_3_ nanostructures with various cycles of ALD Al_2_O_3_ thin film.

**Figure 10:**
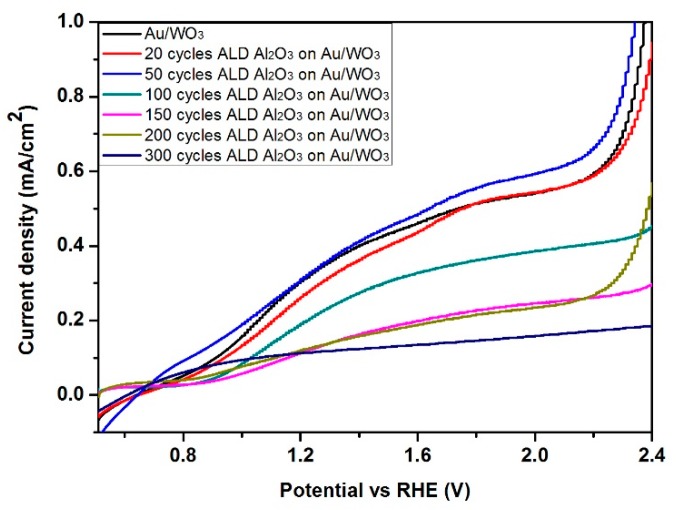
J-V curves of Al_2_O_3_/Au/WO_3_ during photoelectrochemical measurement.

The XPS spectrum of Al 2p shows that the intensity of Al 2p peak increases with the increase of the number of cycles (Figure 9a). In contrast, the intensity of Au 4f peak decreases with the increase of the number of cycles, with very little Au 4f signal intensity remaining after 50 cycles (~6 nm Al_2_O_3_) and no Au signal detected after 150 cycles (~18 nm) (Figure 9b). Our interest in the conformal Al_2_O_3_ is as a barrier/corrosion resistant layer for electrochemistry/photoelectrochemistry. To evaluate the quality of the coating, the photoelectrochemical performance was tested (Figure 10). The results demonstrate that increasing the Al_2_O_3_ film thickness beyond 50 deposition cycles (~6 nm) lead to a significant decrease in photocurrent density, as expected for high dieletric material. Consequently, both the XPS and PEC data indicate that even for a 50 cycles coating, the Al_2_O_3_ film was conformal and without pinholes even on these high aspect ratio, porous nanostructures.

## 4. Conclusions

In the study, the parameters of Al_2_O_3_ thin films atomic layer deposition using aluminum tri-sec-butoxide as a new Al precursor and water for ALD were investigated. The CVD, pulsed CVD and ALD process was optimized, with the best deposition conditions for ALD as follows: ATSB bubbler temperature 120 °C; deposition temperature 200 °C; gas flow rate 120 sccm; ATSB pulse time 2.5 s; Ar purge time 3 min; H_2_O pulse time 2 s; Ar purge time 3 min. A stable ALD process with a growth rate of 0.12–0.15 nm/cycle was observed by measuring the Al_2_O_3_ film thickness on different substrates using ellipsometry. The composition and morphology of the as-synthesized films were analyzed and followed by the comparison of film deposition under different conditions. SEM, AFM and XPS data indicated that the obtained films were dense and continuous with a low concentration of impurities. TEM images of Al_2_O_3_ thin films prepared from ATSB and water on complex nanostructures show uniformity, conformality and good control of thickness, strongly suggesting the potential of using this new ALD precursor for Al_2_O_3_ thin films. These Al_2_O_3_ films are likely to be used as a protection layer on the Au/WO_3_ electrode for photoelectrochemical water splitting.

## Figures and Tables

**Table 1:** Summary of the ALD Al_2_O_3_ thin film deposition parameters and the film thickness on Au/WO_3_, FTO and silicon.

Substrate	Deposition Recipe				Cycle (Number)	Substrate Temperature (°C)	Flow Rate (sccm)	Thickness (nm)
	ATSB	Purge	H_2_O	Purge				
Silicon, FTO and Au/WO_3_	2.5 s	3 min	2 s	3 min	20	200	120	2
					50			7
					100			10
					150			15
					200			20
					300			40

## Supplementary Materials

The following are available online at https://www.mdpi.com/1996-1944/12/9/1429/s1, Figure S1: The relationship between ATSB vapor pressure and ATSB precursor temperature. Figure S2: XRD pattern of Al_2_O_3_ film deposited via CVD. This example was made under condition: precursor temperature 120 °C; deposition temperature 350 °C; gas flow rate 150 sccm; deposition time 24 h. Figure S3: Raman spectra of Al_2_O_3_ film deposited via CVD. This example was made under condition: precursor temperature 120 °C; deposition temperature 350 °C; gas flow rate 150 sccm; deposition time 24 h. Figure S4: Growth rates of the Al_2_O_3_ films as a function of deposition temperature from 200 °C to 300 °C established via ellipsometry. The deposition conditions were 20 s ATSB pulse, 1 min Ar purge, 2 s H_2_O pulse and 3 min Ar pulse for 500 cycles. Figure S5: Refractive index of (a) Al_2_O_3_ [23], and (b) Al_2_O_3_/air (0.757/0.243) Effective Medium Approximation used for ellipsometric modelling of ALD film on glass, Figure 6e. Figure S6: Film thicknesses established via Ellipsometry for ALD growth on Silicon samples (R^2^ fit quality for each ellipsometric fitting labelled). EMA concentration for all samples held at 74.5%. ATSB pulse duration 2.5 s. Table S1: Table of structural parameters from for ellipsometric model fitting between (1.25–5 eV) for samples produced via ALD (250 cycles: ATSB pulse indicated, 3 min purge, 2 s H_2_O pulse, 3 min purge).
