# Supplementary material for: Use of a New Non-Pyrophoric Liquid Aluminum Precursor for Atomic Layer Deposition
Source: Materials (Basel). 2019 May 2;12(9):1429. doi: 10.3390/ma12091429 (PMC6540254; doi:10.3390/ma12091429)
Supplement: Supplementary file 1 [file materials-12-01429-s001.pdf]

materials

Supplementary Materials

Use of a New Non-Pyrophoric Liquid Aluminium Precursor for Atomic Layer Deposition

Xueming Xia [1], Alaric Taylor [2], Yifan Zhao [3], Stefan Guldin [2] and Chris Blackman [1,*]

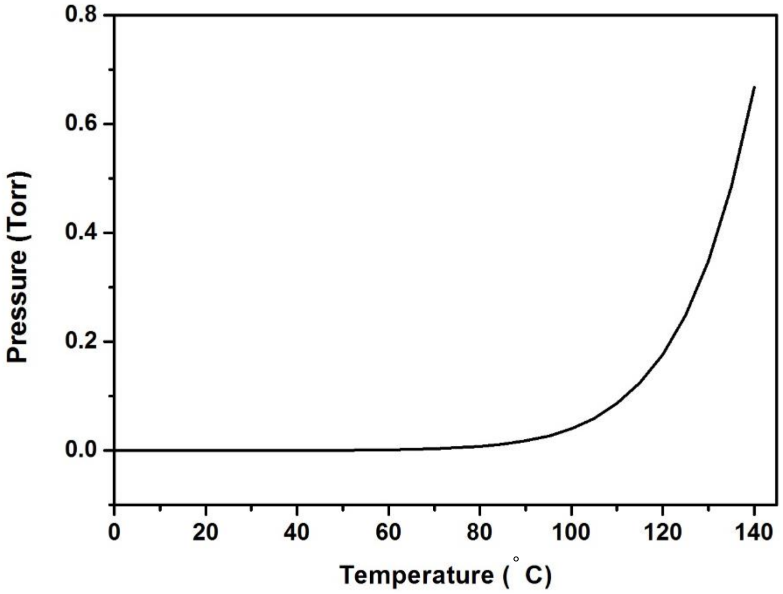

Figure S1. The relationship between ATSB vapour pressure and ATSB precursor temperature.

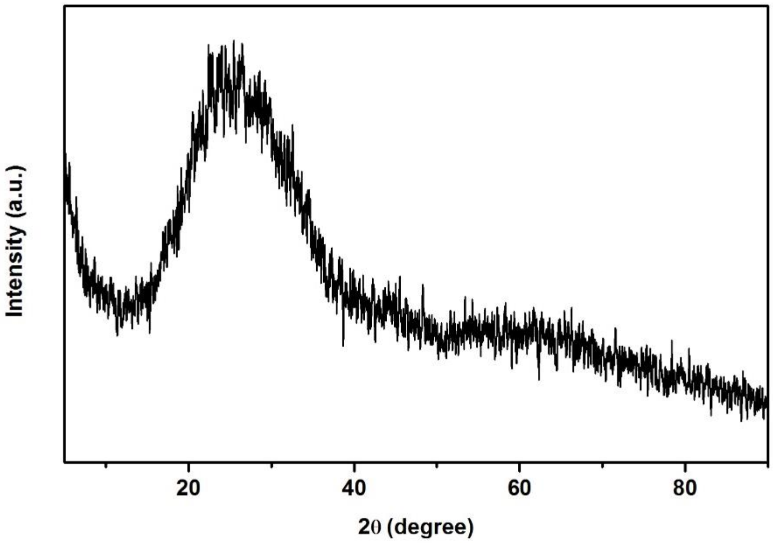

Figure S2. XRD pattern of Al_2O_3 film deposited via CVD. This example was made under condition: precursor temperature 120 °C; deposition temperature 350 °C; gas flow rate 150 sccm; deposition time 24 h.

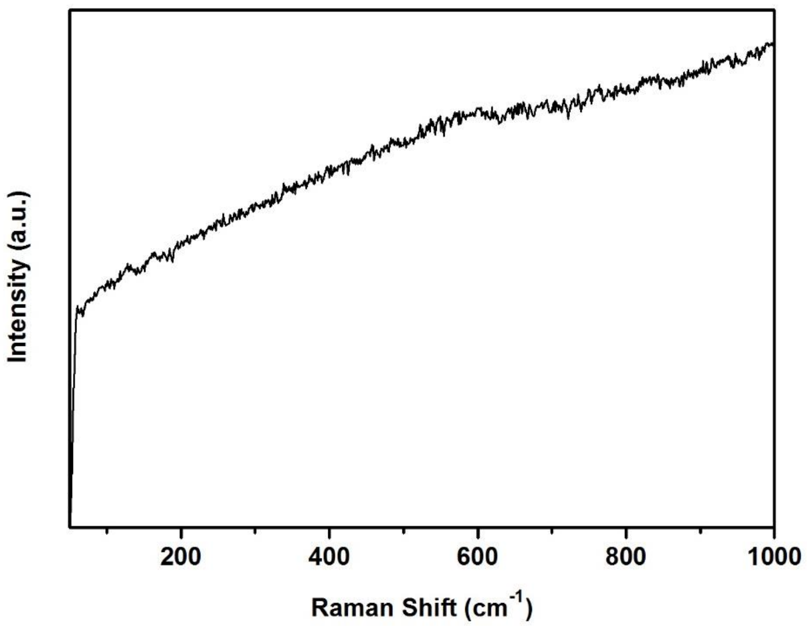

Figure S3. Raman spectra of Al_2O_3 film deposited via CVD. This example was made under condition: precursor temperature 120 °C; deposition temperature 350 °C; gas flow rate 150 sccm; deposition time 24 h.

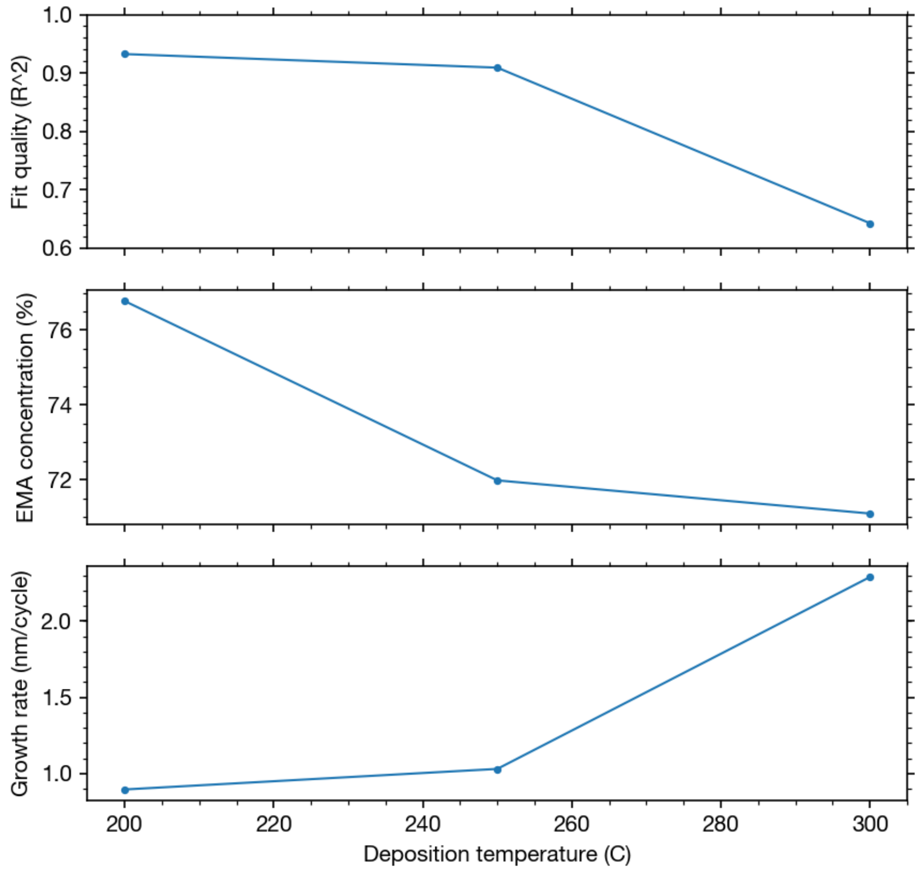

Figure S4. Growth rates of the Al_2O_3 films as a function of deposition temperature from 200 °C to 300 °C established via ellipsometry. The deposition conditions were 20 s ATSB pulse, 1 min Ar purge, 2 s H_2O pulse and 3 min Ar pulse for 500 cycles.

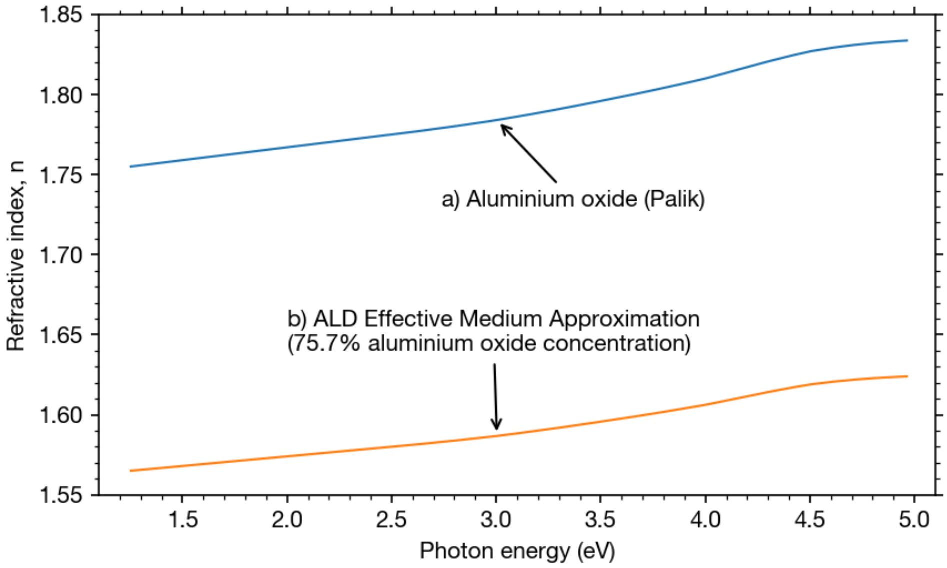

Figure S5. Refractive index of (**a**) Al_2O_3 [23], and (**b**) Al_2O_3/air (0.757/0.243) Effective Medium Approximation used for ellipsometric modelling of ALD film on glass, Figure 6e.

Table S1. Table of structural parameters from for ellipsometric model fitting between (1.25–5 eV) for samples produced via ALD (250 cycles: ATSB pulse indicated, 3 min purge, 2 s H_2O pulse, 3 min purge).

AFM Shown (Figure)	Substrate	ATSB Dose (s)	Layer Thickness (nm)	EMA Concentration (%)	Refractive Index @ 632.8 nm	Fit Quality, R^2	Growth Rate (nm/cycle)
6a & b	silicon	2.5	29.8	74.5	1.56	0.997	0.12
6c & d	quartz	2.5	37.7	74.2	1.56	0.965	0.15
6e & f	glass	2.5	31.2	75.7	1.57	0.916	0.12
-	glass	20	257.8	72.0	1.54	0.909	1.03

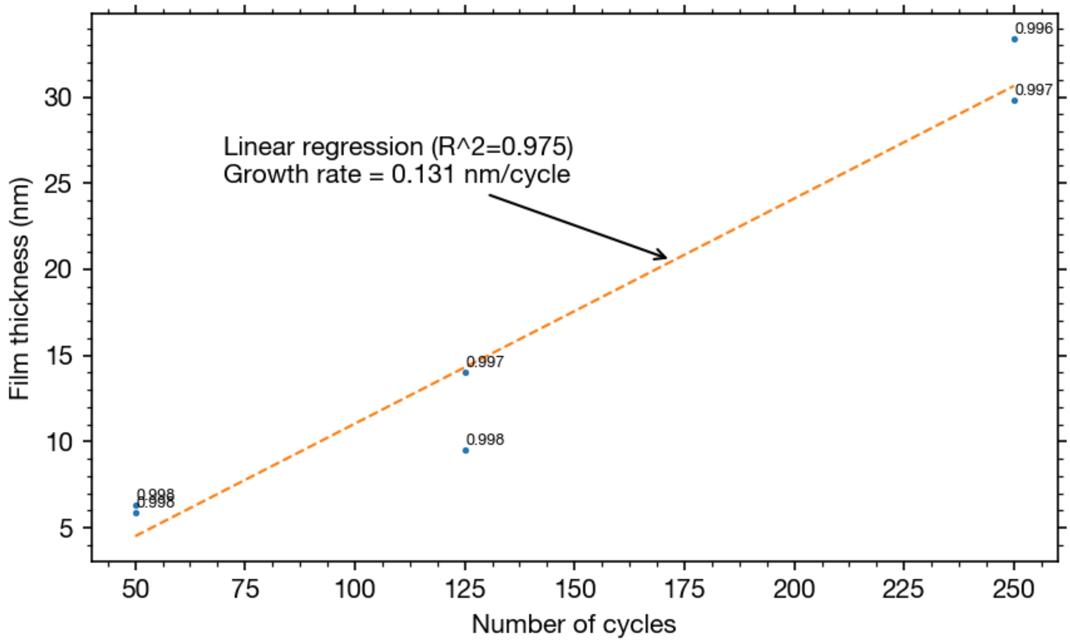

Figure S6. Film thicknesses established via Ellipsometry for ALD growth on Silicon samples (R^2 fit quality for each ellipsometric fitting labelled). EMA concentration for all samples held at 74.5%. ATSB pulse duration 2.5 s.

© 2019 by the authors. Licensee MDPI, Basel, Switzerland. This article is an open access article distributed under the terms and conditions of the Creative Commons Attribution (CC BY) license (http://creativecommons.org/licenses/by/4.0/).
